# Association of inflammatory blood markers and pathological complete response in HER2-positive breast cancer: a retrospective single-center cohort study

**DOI:** 10.3389/fimmu.2024.1465862

**Published:** 2024-11-19

**Authors:** Xiaobin Chen, Qindong Cai, Lin Deng, Minyan Chen, Min Xu, Lili Chen, Yuxiang Lin, Yan Li, Yali Wang, Hanxi Chen, Shunyi Liu, Jinqiao Wu, Xin Tong, Fangmeng Fu, Chuan Wang

**Affiliations:** ^1^ Department of Breast Surgery, Fujian Medical University Union Hospital, Fuzhou, Fujian, China; ^2^ Department of General Surgery, Fujian Medical University Union Hospital, Fuzhou, Fujian, China; ^3^ Breast Cancer Institute, Fujian Medical University, Fuzhou, Fujian, China; ^4^ Department of General Surgery, Fuzong Clinical Medical College of Fujian Medical University, Fuzhou, China; ^5^ Department of General Surgery, The 900^th^ Hospital of Joint Logistic Support Force, Fuzhou, China; ^6^ Department of Thyroid and Breast Surgery, The Third Hospital of Xiamen, Xiamen, China

**Keywords:** HER2 positive breast cancer, neoadjuvant response, monocyte-to-lymphocyte ratio, neutrophil-to-lymphocyte ratio, platelet-to-lymphocyte ratio

## Abstract

**Introduction:**

The association between inflammatory blood markers (IBMs) (monocyte-to-lymphocyte ratio [MLR], neutrophil-to-lymphocyte ratio [NLR], and platelet-to-lymphocyte ratio [PLR]) and breast cancer has been extensively studied. However, the predictive role of IBMs in the neoadjuvant response of human epidermal growth factor receptor 2 (HER2)-positive breast cancer remains unclear.

**Methods:**

This study included 744 patients with HER2 positive breast cancer treated with neoadjuvant therapy. Baseline MLR, NLR, and PLR data were collected to investigate the association between IBMs and pathological complete response (pCR).

**Results:**

MLR, NLR, and PLR were not associated with neoadjuvant response in the overall population before and after matching. Subgroup analysis stratified by neoadjuvant therapy suggested that these IBMs play a diverse predictive role in response to chemotherapy alone and chemotherapy plus anti-HER2 therapy. A high MLR and NLR, but not PLR, were associated with lower pCR rates in HER2-targeted therapy (MLR: OR=0.67, *P*=0.023; NLR: OR=0.665, *P*=0.02; PLR: OR=0.801, *P*=0.203). Among the anti-HER2 treatment population, patients with a high MLRs (pCR rate, 40.2%) could be divided into MLR^high^/NLR^high^ (pCR rate, 36.3%) and MLR^high^/NLR^low^ (pCR rate, 48.9%) groups when the NLR was considered. The pCR rates of the MLR^high^/NLR^low^ and low-MLR groups were similar (pCR rate, 47.6%). A comparable stratification effect was observed in patients with high NLR.

**Conclusions:**

IBMs play a diverse predictive role in pCR in HER2-positive breast cancer stratified by neoadjuvant regimens. The combination of high MLR and high NLR enabled better identification of patients with poor responses to anti-HER2 therapy than high MLR or NLR alone.

## 1 Introduction

Human epidermal growth factor receptor 2 (HER2)-positive breast cancer is characterized by the overexpression/amplification of *C-erbB2*, accounting for 15%–25% of newly diagnosed breast cancers (1). Based on hormone receptor (HR) status, HER2-positive breast cancer can be further divided into HER2+/HR- and HER2+/HR+ (Luminal B, HER2 positive) breast cancer (2). HER2 positive breast cancer is associated with an aggressive phenotype and poor prognosis (3). In the past two decades, the advent of anti-HER2 agents has significantly improved pathological complete response (pCR) rates and the prognosis of HER2-positive breast cancer (1, 4). However, the response to HER2-targeted therapy varies among individuals (5). Substantial efforts have been made to explore the predictors of pCR for HER2-positive breast cancer, as pCR is commonly used as a surrogate for good response and favorable long-term survival (6–8). However, the clinical need to identify readily available factors that can accurately predict pCR for HER2-positive breast cancer remains.

The status of the host immune system is known to play an important role in influencing the therapeutic response and outcome of HER2-positive breast cancer (9). The effect of the immune system was more reasonable for patients treated with trastuzumab or trastuzumab plus pertuzumab, as antibody-dependent cellular cytotoxicity (ADCC) is one of the most important mechanisms of the antitumor action of these drugs (10). For example, higher baseline tumor-infiltrating lymphocytes (TILs) are associated with increased pCR rates in HER2-positive breast cancer treated with neoadjuvant targeted therapy (11). In addition, omics-based studies have also suggested that the high expression of immune signature-related genes is associated with a better response to anti-HER2 therapy (12, 13). However, evaluation of TILs or other tissue-based immune-related parameters has not been routinely performed in clinical practice. Thus, more accessible immune indicators that predict neoadjuvant response in HER2-positive breast cancer are needed.

The status of the host immune system can also be reflected by peripheral inflammatory blood markers (IBMs), such as monocyte-to-lymphocyte ratio (MLR), neutrophil-to-lymphocyte ratio (NLR), and platelet-to-lymphocyte ratio (PLR) (14). A strong link between these IBMs and breast cancer has been previously validated. For instance, a high NLR or PLR is associated with a lower pCR rate among patients with triple-negative and luminal-like (HER2-) breast cancer (15–17). For HER2-positive breast cancer, high NLR, MLR, and PLR have been demonstrated to be associated with poor prognosis in both adjuvant and metastatic settings (18–21). However, the possible predictive value of baseline MLR, NLR, and PLR for pCR in HER2-positive breast cancer treated with neoadjuvant targeted therapy remains unknown.

Hence, we conducted this single-center retrospective study to investigate the association of baseline MLR, NLR, and PLR with pCR in HER2-positive breast cancer treated with trastuzumab- or trastuzumab plus pertuzumab-based neoadjuvant therapy. Furthermore, cases of HER2-positive breast cancer treated with neoadjuvant chemotherapy without anti-HER2 agents were also included to explore whether the predictive value of IBMs was dependent on anti-HER2 therapy.

## 2 Methods

### 2.1 Patient selection

In this single-center retrospective study, 767 patients with HER2-positive breast cancer treated with neoadjuvant therapy were recruited from the Fujian Medical University Union Hospital between June 2012 and December 2023. Patients were excluded from the study if they were diagnosed with metastatic disease, acute or chronic inflammation, hematological diseases, or if baseline IBMs were unavailable (n=23). This study was conducted in compliance with the Declaration of Helsinki. The study protocol was approved by the Ethics Committee of the Fujian Medical University Union Hospital. All participants provided written informed consent before inclusion in the study.

### 2.2 Data collection

Routine peripheral blood examination results were obtained before the initiation of neoadjuvant therapy. NLR was calculated by dividing the absolute number of neutrophils by the absolute lymphocyte counts (ALCs), MLR was calculated by dividing the absolute number of monocytes by ALCs, and PLR was determined as the ratio between the absolute count of platelets and ALCs. Clinicopathological features, including age, body mass index (BMI), baseline tumor size (evaluated using color Doppler ultrasound), axillary lymph node status (evaluated using color Doppler ultrasound), hormone receptor (HR) status, Ki67 expression, and neoadjuvant response, were retrospectively collected.

### 2.3 Neoadjuvant treatments

All patients received neoadjuvant chemotherapy, with or without HER2-targeted therapy. For patients who received chemotherapy alone, the regimen consisted of EC-T (epirubicin 100 mg/m^2^ and cyclophosphamide 600 mg/m^2^ every three weeks for four cycles, followed by docetaxel 80 mg/m^2^ for four cycles). For HER2 targeted population, patients were treated with the following regimens: 1) EC-T plus targeted therapy (epirubicin 100 mg/m^2^ and cyclophosphamide 600 mg/m^2^ every three weeks for four cycles, followed by docetaxel 80 mg/m^2^ and targeted therapy every three weeks for four cycles) and 2) TCb plus targeted therapy (docetaxel 75 mg/m^2^, carboplatin [area under curve = 6], and targeted therapy every three weeks for six cycles). Trastuzumab (initiated with a loading dose of 8 mg/kg, followed by a maintenance dose of 6 mg/kg) or trastuzumab plus pertuzumab (840 mg as the loading dose in cycle 1 and 420 mg thereafter every three weeks) was administered to patients who received targeted therapy.

### 2.4 Pathological evaluation

The statuses of estrogen receptor (ER), progesterone receptor (PR), HER2 expression, fluorescence *in situ* hybridization (FISH), and Ki67 expression were obtained from core needle biopsy specimens before neoadjuvant therapy. Tumors that were determined to be HER2 3+ by immunohistochemistry (IHC) or HER2 2+ with HER2 amplification as assessed by FISH were defined as HER2-positive. ER and PR were categorized as positive if ≥1% of invasive tumor nuclei in the samples were positive. HR was classified as positive when ER or PR was positive by IHC. Pathological complete response was defined as the absence of residual invasive disease in the breast and the axillary lymph nodes, the presence of carcinoma *in situ* is allowed (ypT0/is ypN0).

### 2.5 Statistical analysis

A median age of 48 years was used to categorize the patients into two groups. Median Ki67 was used to divide patients into a low Ki67 group (≤40%) and high Ki67 group (>40%) (8, 22). Patients were categorized into underweight (BMI of <18.5 kg/m^2^), normal weight (BMI of 18.5 to <24 kg/m^2^), overweight (BMI of 24 to <28 kg/m^2^), and obesity (BMI of ≥28 kg/mg^2^) according to the Working Group on Obesity in China (WGOC) definition (23). A t-test was used to calculate the statistical differences in the distributions of MLR, NLR, and PLR across the different groups. The chi-square test was used to explore the association between high/low IBMs and clinicopathological factors.

Propensity score matching (PSM) and inverse probability of treatment weighting (IPTW) were applied to adjust for differences in baseline characteristics. The propensity score was calculated by multivariable logistic analysis with the IBMs as the objective variables and clinical characteristics (including age, BMI, clinical T stage, nodal status, hormone receptor, HER2 staining intensity, Ki67 [%], and neoadjuvant therapy) as the explanatory variables. PSM was performed using the nearest neighbor-matching with caliper values of 0.02 for 1:1 matching. We assigned a weight of 1/PS to patients with high IBMs and a weight of 1/(1−PS) to patients with low IBMs. Multivariable logistic regression analysis was performed to investigate the predictive value of IBMs for pCR. Statistical significance was set at *P* < 0.05. All statistical analyses were performed using R software (version 4.2.3) and SPSS (version 26.0).

## 3 Results

### 3.1 Baseline clinicopathological characteristics

**Figure 1:**
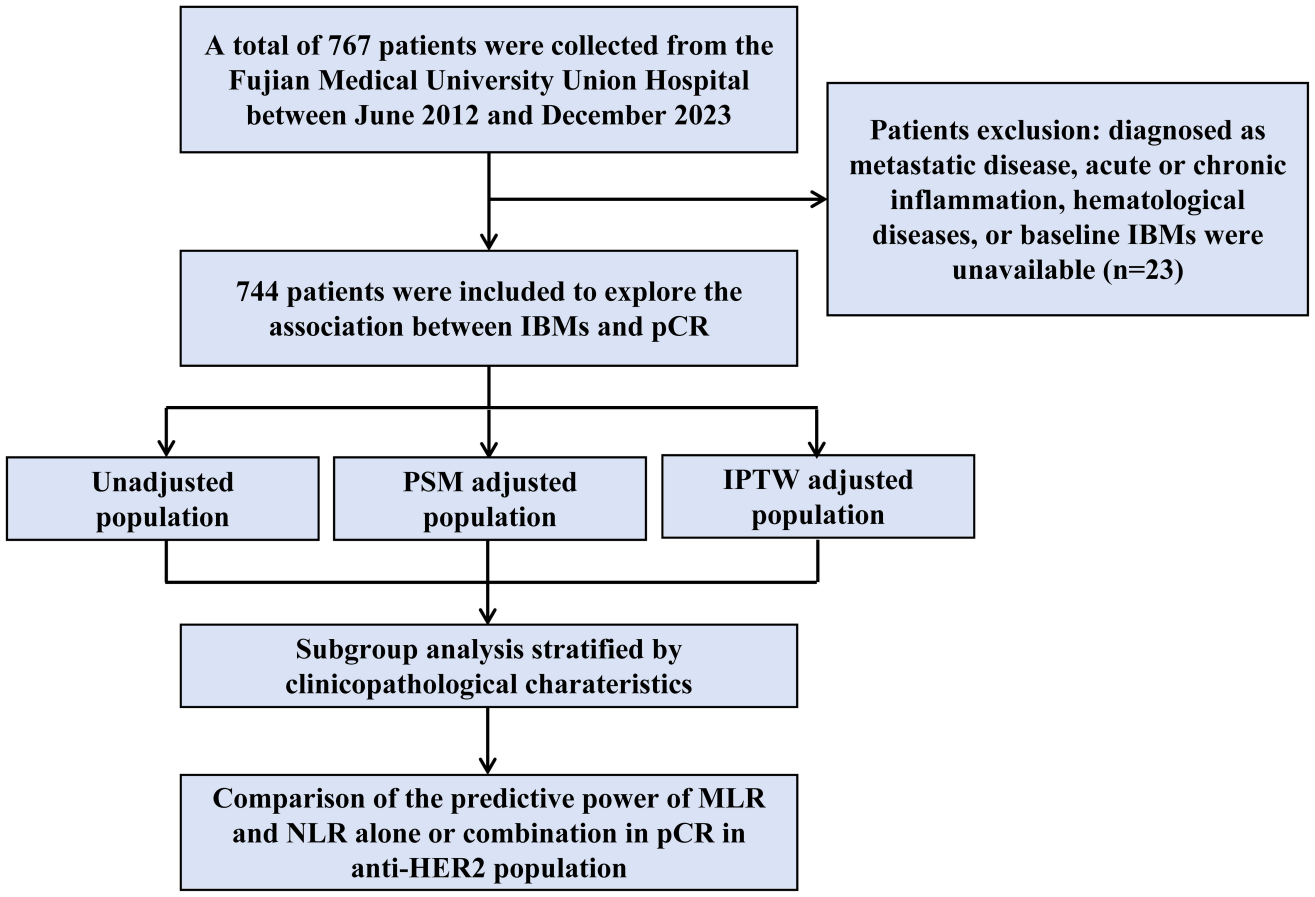
Schematic representation of the patient selection process. A total of 767 patients were enrolled into our study, and the data of 744 patients were included in the final cohort analysis. IBMs, inflammatory blood markers; pCR, pathological complete response; PSM, propensity score matching; IPTW, inverse probability of treatment weighting. MLR, monocyte-to-lymphocyte ratio; NLR, neutrophil-to-lymphocyte ratio.

The study flowchart is shown in **Figure 1**. Data from 744 patients with HER2-positive breast cancer treated with neoadjuvant therapy were retrospectively collected (**Table 1**). A total of 135 patients received neoadjuvant chemotherapy without anti-HER2 therapy, whereas 609 received chemotherapy plus trastuzumab-based anti-HER2 therapy (trastuzumab or trastuzumab plus pertuzumab).

**Table 1:** Baseline clinicopathological characteristics of patients.

Characteristics	Patients (n)	Percentage
**Total**	744	100%
Age (years)		
≤48	384	51.61%
>48	360	48.39%
BMI (kg/m^2^)		
Under/Normal weight	459	61.69%
Overweight/Obesity	285	38.31%
Clinical T stage		
T1-2	574	77%
T3-4	170	23%
Nodal status		
Negative	225	30.24%
Positive	519	69.76%
Hormone receptor		
Negative	349	46.91%
Positive	395	53.09%
HER2 staining intensity		
2+ and FISH +	81	10.89%
3+	663	89.11%
Ki67 (%)		
≤40	408	54.84%
>40	336	45.16%
Neoadjuvant therapy		
Chemotherapy	135	18.15%
Chemo+targeted therapy	609	81.85%
Neoadjuvant response		
pCR	288	38.71%
non-PCR	456	61.29%

BMI, body mass index; HER2, human epidermal growth factor receptor 2; pCR, pathological complete response.

### 3.2 The association between IBMs and clinicopathological factors

When considering IBMs as continuous variables, MLR was significantly higher in patients treated with chemotherapy alone than in those treated with chemotherapy plus anti-HER2 therapy (**Supplementary Figure S1**). Furthermore, cN-negative patients tended to have higher MLRs; however, the difference was not statistically significant (*P*=0.051). The distributions of NLR (**Supplementary Figure S2**) and PLR (**Supplementary Figure S3**) were similar among the different subgroups. Although patients with HER2 2+ breast cancer were more likely to have higher NLRs than those with HER2 3+ breast cancer (*P*=0.06), a trend toward lower PLRs was demonstrated for overweight/obese patients, compared with underweight/normal weight patients (*P*=0.055). No other clinicopathological factors were associated with the IBMs.

MLRs, NLRs, and PLRs were then divided into low and high groups according to their median values. MLR was significantly associated with neoadjuvant therapy (*P*<0.001) and nodal status (*P=*0.046) when included as categorical variables (**Supplementary Table S1**). The baseline characteristics of the high and low MLR subgroups were well balanced after adjustment for PSM or IPTW (**Supplementary Tables S1**, **S2**), as well as NLR and PLR (**Supplementary Tables S3**–**S6**).

### 3.3 MLR, NLR, and PLR play no predictive role for pCR in the overall population

In the raw data of the overall population, none of the three IBMs were independent predictive factors for pCR in the total population after adjusting for clinicopathological factors, including age, BMI, clinical T stage, nodal status, hormone receptor, HER2 staining intensity, Ki67 (%), and neoadjuvant therapy (**Table 2**, **Supplementary Tables S7**–**S9**). Similar results were observed in the PSM- and IPTW-adjusted populations (**Table 2**, **Supplementary Tables S7**–**S9**).

**Table 2:** Association of IBMs and pCR in Multivariable analysis.

	Unweighted population			PS weighted population			IPTW weighted population		
Characteristics	OR	95%CI	*P* value#	OR	95%CI	*P* value#	OR	95%CI	*P* value#
MLR									
Low	1 (reference)			1 (reference)			1 (reference)		
High	0.802	0.580-1.108	0.18	0.811	0.573-1.149	0.239	0.813	0.589-1.124	0.211
NLR									
Low	1 (reference)			1 (reference)			1 (reference)		
High	0.787	0.570-1.085	0.144	0.789	0.565-1.101	0.163	0.789	0.573-1.088	0.148
PLR									
Low	1 (reference)			1 (reference)			1 (reference)		
High	0.939	0.681-1.294	0.699	0.981	0.704-1.368	0.909	0.938	0.681-1.291	0.694

#Adjusted for age, BMI, clinical T stage, nodal status, hormone receptor, HER2 staining intensity, Ki67 (%), neoadjuvant therapy.

BMI, body mass index; CI, confidence interval; IBMs, inflammatory blood markers; MLR, monocyte-to-lymphocyte ratio; NLR, neutrophil-to-lymphocyte ratio; OR, odds ratio; PLR, platelet-to-lymphocyte ratio; HER2, human epidermal growth factor receptor 2.

### 3.4 The predictive value of IBMs is diverse in subgroups stratified by neoadjuvant therapy

**Figure 2:**
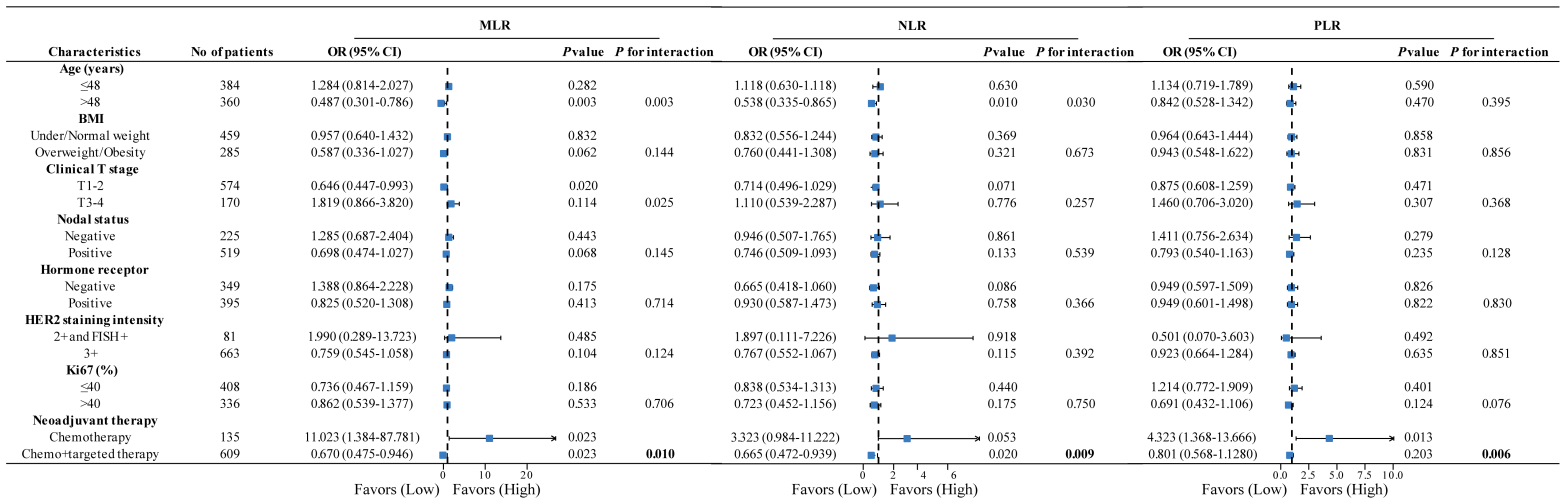
Forest plots of subgroup analysis stratified by clinicopathological factors to explore the association between IBMs and pCR among different populations. *P* value and *P* for interaction was adjusted for age, BMI, clinical T stage, nodal status, hormone receptor, HER2 staining intensity, Ki67 (%), and neoadjuvant therapy (exception for stratification factor). IBMs, inflammatory blood markers; pCR, pathological complete response; BMI, body mass index; HER2, human epidermal growth factor receptor 2.

Owing to the ambiguous predictive value of IBMs in neoadjuvant response prediction among the overall population, a subgroup analysis was conducted to investigate the role of IBMs in different subgroups. In the subgroup analysis by neoadjuvant therapy, high MLRs were more likely to achieve pCR than those with low MLRs (OR=11.023, *P*=0.023) among patients treated with chemotherapy alone. Notably, this was not the case for patients treated with chemotherapy plus anti-HER2 therapy; the opposite trend was found in this population (OR=0.67, *P*=0.023), resulting in a statistically significant interaction between MLR and neoadjuvant therapy (*P* for interaction=0.01) (**Figure 2**). Interestingly, a similar tendency was observed for NLR and PLR; the interaction between neoadjuvant therapy and NLR (*P* for interaction=0.009) or PLR (*P* for interaction=0.006) was also significant (**Figure 2**). These findings suggest that the predictive value of IBMs is diverse in subgroups stratified by neoadjuvant therapy.

### 3.5 The combination of high MLR and high NLR may better identify patients with poor responses to anti-HER2 therapy than high MLR or high NLR alone

Overall, these results suggest that MLR (OR=0.67, *P*=0.023) and NLR (OR=0.6765, *P*=0.02) were significantly associated with pCR, but not PLR (OR=0.801, *P*=0.203) in patients treated with HER2-targeted therapy; hinting that MLR and NLR might be more valuable than PLR in current clinical practice. Given that host immunity status might be better reflected by the combination of IBMs, the predictive value of MLR combined with NLR for pCR was analyzed. A total of 744 patients were divided into three groups according to MLR and NLR status: MLR^low^/NLR^low^ (n=258), MLR^high^/NLR^low^ or MLR^low^/NLR^high^ (n=228), and MLR^high^/NLR^high^ (n=258). The combination of MLR and NLR did not predict pCR in the overall population (**Supplementary Table S10**). However, when stratified by neoadjuvant therapy, MLR^high^/NLR^high^ was significantly associated with a lower pCR rate than MLR^low^/NLR^low^ in patients treated with anti-HER2 therapy (OR=0.548, *P*=0.005) (**Table 3**, **Supplementary Tables S11**, **S12**). The pCR rate of the MLR^high^/NLR^low^ and MLR^low^/NLR^high^ group was similar to that of the MLR^low^/NLR^low^ group (OR=1.077, *P*=0.728).

**Table 3:** The association between MLR/NLR and pCR stratified by neoadjuvant therapy.

			Adjusted Multivariable analysis	
Neoadjuvant therapy	N	pCR rate (%)	OR (95% CI)	*P* value^#^
Chemotherapy population				
MLR/NLR (Low/Low)	31	1 (3.2)	1 (reference)	
MLR/NLR (High/Low or Low/High)	39	3 (7.7)	2.554 (0.242-26.992)	0.436
MLR/NLR (High/High)	65	15 (23.1)	9.528 (1.154-78.638)	0.036
Trend			3.316 (1.330-8.265)	0.01
Chemo+targeted therapy population				
MLR/NLR (Low/Low)	227	108 (47.5)	1 (reference)	
MLR/NLR (High/Low or Low/High)	189	91 (48.2)	1.077 (0.708-1.638)	0.728
MLR/NLR (High/High)	193	70 (36.2)	0.548 (0.361-0.832)	0.005
Trend			0.748 (0.608-0.920)	0.006
*P* for interaction				0.001

^#^Adjusted for age, BMI, clinical T stage, nodal status, hormone receptor, HER2 staining intensity, Ki67 (%). BMI, body mass index; CI, confidence interval; MLR, monocyte-to-lymphocyte ratio; NLR, neutrophil-to-lymphocyte ratio; OR, odds ratio; PLR, platelet-to-lymphocyte ratio; HER2, human epidermal growth factor receptor 2.

**Figure 3:**
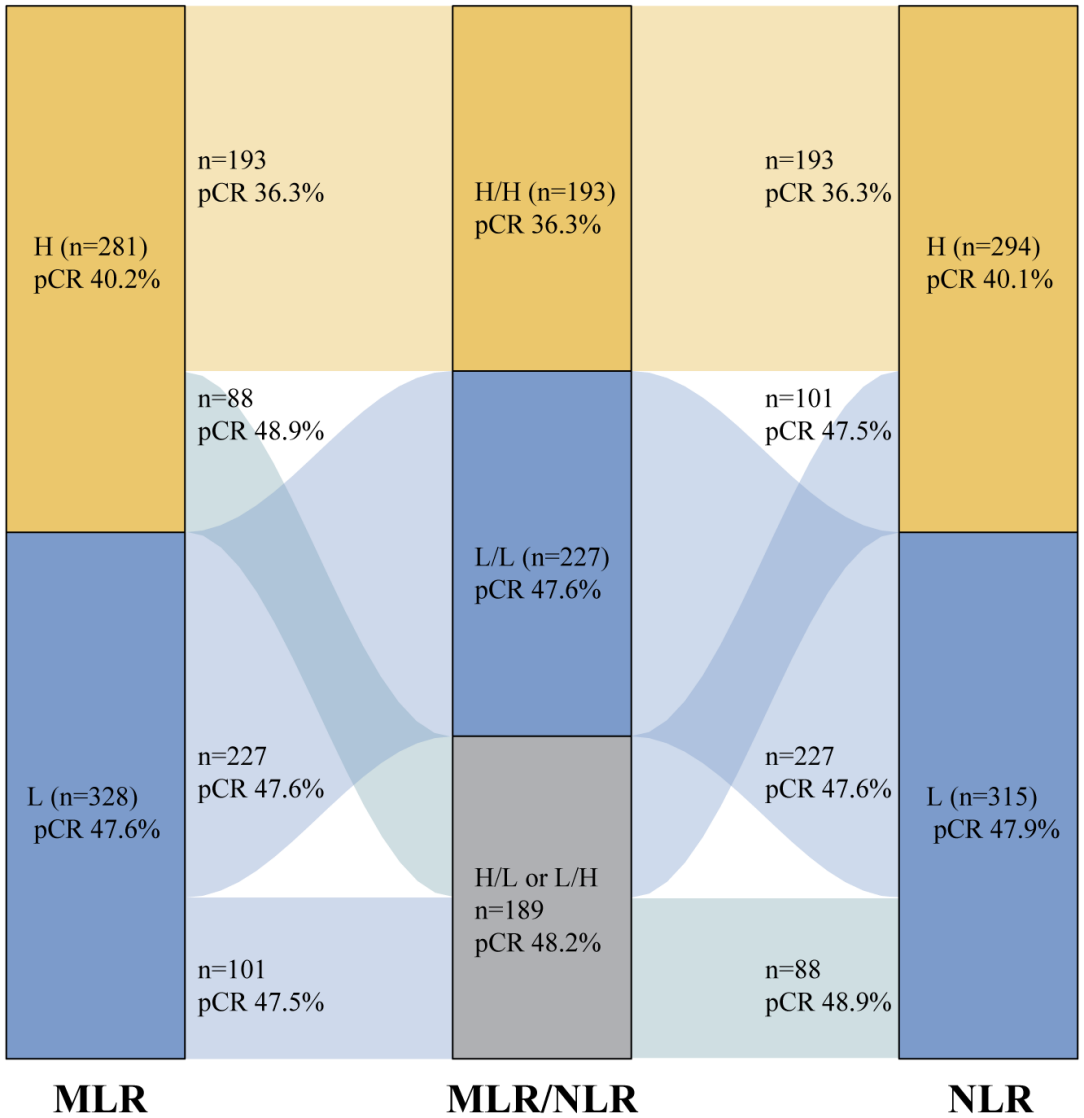
The comparison of the predictive value of MLR and NLR alone or combination in pCR in the anti-HER2 cohort; H=High; L=Low. MLR, monocyte-to-lymphocyte ratio; NLR, neutrophil-to-lymphocyte ratio; pCR, pathological complete response; HER2, human epidermal growth factor receptor 2.

The predictive values of MLR and NLR alone or combination for pCR in the anti-HER2 cohort were compared. A total of 281 patients were characterized as having high MLRs (pCR rate, 40.2%). Of these patients, 88 were allocated to the MLR^high^/NLR^low^ group (pCR rate, 48.9%), and the remaining were allocated to the MLR^high^/NLR^high^ group (n=193; pCR rate, 36.3%) (**Figure 3**). The pCR rate of MLR^high^/NLR^low^ group was significantly higher than that of the MLR^high^/NLR^high^ group (OR=1.679, *P*=0.047). Interestingly, no difference was observed between the MLR^high^/NLR^low^ and low-MLR groups (pCR rate, 47.6%). For the high-NLR population, the pCR rate of the NLR^high^/MLR^low^ group (pCR rate, 47.5%) was higher than that of the NLR^high^/MLR^high^ group (pCR rate, 36.3%) (OR=1.591, *P*=0.062) but similar to that of the low-NLR group (pCR rate, 47.9%) (**Figure 3**). These data suggest that the combination of MLR and NLR can better identify patients with poor responses to anti-HER2 therapy than either IBM alone.

## 4 Discussion

In this study, we explored the association between IBMs (MLR, NLR, and PLR) and neoadjuvant therapeutic response in HER2-positive breast cancer (n=744). Among the overall population, MLR, NLR, and PLR were not independent predictors of pCR. In the subgroup analysis stratified by neoadjuvant therapy, an opposite role of IBMs in neoadjuvant response prediction was observed between the subgroups. In addition, the combination of high MLR and NLR better identified patients with poor responses to anti-HER2 therapy than high MLR or NLR alone.

The role of IBMs in HER2-positive breast cancer has been previously reported. In a study focused on HER2-positive advanced breast cancer treated with trastuzumab-pertuzumab therapy, a high baseline pan-immune-inflammation value (PIV) was associated with poor prognosis, rather than MLR, NLR, and PLR (24). Exploratory analysis of data from the CLEOPATRA trial revealed that a low baseline NLR was associated with better survival outcomes in patients treated with docetaxel plus trastuzumab, with or without pertuzumab (25). However, the roles of MLR, NLR, and PLR in the prediction of neoadjuvant response in HER2-positive breast cancer have not been well investigated. To the best of our knowledge, only one study has explored the association between preoperative systemic inflammation response index (SIRI) and pCR in HER2-positive breast cancer (26). In the aforementioned study, 240 patients with HER2-positive breast cancer treated with chemotherapy plus HER2-targeted therapy were included. SIRI was the only variable that predicted pCR with statistical significance; NLR, PLR, and LMR did not display predictive value. Considering the limited sample size of this study, the predictive values of MLR, NLR, and PLR for neoadjuvant response in HER2-positive breast cancer are still worth studying.

In our study, a relatively large number of patients with HER2-positive breast cancer treated with neoadjuvant therapy were included (n=744). MLR, NLR, and PLR were not found to be independent predictive factors for pCR in the total population. However, when stratified by neoadjuvant therapy, a high MLR was less likely to achieve pCR than a low MLR in the anti-HER2 population (OR=0.670, *P*=0.023). Interestingly, this tendency was also observed in NLR (OR=0.665, *P*=0.02), but not in PLR (OR=0.801, *P*=0.203).

MLR and NLR are cost-effective, readily available IBMs that reflect the systemic immune status of the host (27). Lymphocytes play important roles in immune surveillance and the inhibition of tumor cell proliferation and migration, and elevated infiltration of lymphocytes in tumors has been associated with a better anti-HER2 response (28, 29). In contrast, an *in vivo* study suggested that blood monocytes promote tumor growth by differentiating into tolerogenic dendritic cells (DCs) that produce interleukin-10 (IL-10) and potently induce regulatory T cell responses (30). Furthermore, circulating monocytes were recruited into primary tumors and the metastatic niche, and differentiation into M2 phenotype tumor-associated macrophages (TAMs) under the induction of chemokines, including IL-10 and transforming growth factor-β (TGF-β), result in an immunosuppressive tumor microenvironment (31, 32). Neutrophils are also known to correlate with pro-tumor activity, accelerate tumor cell proliferation, and promote the metastatic potential of tumor cells (33). High NLR has been significantly associated with lower tumor-infiltrating lymphocytes (TILs) and correlated with a higher proportion of FOXP3+ T-cells in TILs (34). Moreover, neutrophils inhibit the cytotoxic activity of lymphocytes and natural killer cells, leading to an immunosuppressive state (35, 36).

The tumor immune microenvironment plays an important role in modulating the response to anti-HER2 therapy, as the antitumor effects of trastuzumab and pertuzumab partially rely on ADCC (9, 10). ADCC might be suppressed in patients with high MLRs or NLRs owing to their immunosuppressive states, leading to poor responses to trastuzumab therapy. Hence, it is biologically plausible that a higher MLR or NLR might indicate an inferior neoadjuvant response to HER2-targeted therapy in HER2-positive breast cancer.

In addition to patients with HER2-positive breast cancer treated with anti-HER2 therapy, those treated with neoadjuvant chemotherapy alone were also included. Although the sample size was limited (n=135), a significant association between the IBMs and pCR was observed. Interestingly, high IBMs were associated with better neoadjuvant responses in this population, in contrast to those in the HER2-targeted therapy population, resulting in a significant interaction between neoadjuvant therapy and IBMs. The role of the immune system in the chemotherapy treated HER2 positive breast cancer is unclear because the combination of chemotherapy and anti-HER2 therapy has become the standard treatment for these patients. Nevertheless, an exploratory analysis of the FinHER trial cohort revealed that each 10% increase in TILs was associated with improved prognosis in patients randomized to the trastuzumab arm. Meanwhile, for every 10% increase in TILs, the incidence of DDFS increased by 1.22 times in the chemotherapy arm for HER2-positive breast cancer (37). The interaction between immune status and treatment group (chemotherapy or chemotherapy plus trastuzumab) was also observed in the North Central Cancer Treatment Group N9831 Adjuvant Trastuzumab Trial population (38). These results further suggested that the predictive value of IBMs for pCR in HER2-positive breast cancer is dependent on the type of treatment received.

In the present study, the combination of high MLR and NLR was found to be superior to high MLR or NLR alone for the identification of HER2-positive breast cancer that showed poor response to neoadjuvant HER2-targeted therapy. The pCR rate for the high-MLR group was 40.2%. Patients with high MLRs can be divided into the MLR^high^/NLR^low^ (pCR rate, 48.9%) and MLR^high^/NLR^high^ (pCR rate, 36.3%) groups based on NLR status. The pCR rate of the MLR^high^/NLR^low^ group was significantly higher than that of the MLR^high^/NLR^high^ group (OR=1.679, *P*=0.047) but showed no difference when compared with the low-MLR group (pCR rate, 47.5%). Patients with high NLRs can also be divided into two cohorts based on MLR, which demonstrates a dramatic difference in the anti-HER2 response. These data indicate that the combination of MLR and NLR might better reflect host immune status than MLR or NLR alone, as an immunosuppressive phenotype, instead of a single IBM, is involved in the neoadjuvant anti-HER2 response (39).

Our study had some strengths and weaknesses. First, we included a relatively large sample size of 744 patients with HER2-positive breast cancer, which strengthened the reliability of our results. Secondly, patients with HER2-positive breast cancer treated with neoadjuvant chemotherapy alone were included in our study, which helped us to better understand the predictive role of IBMs in HER2-positive breast cancer. However, this was a single-institution retrospective study with the possibility of bias. Furthermore, the lack of prognostic information hindered exploration of the association between IBMs and prognosis.

## 5 Conclusion

In this single-center study, we found diverse predictive roles of MLR, NLR, and PLR in the neoadjuvant response of HER2-positive breast cancer. High MLR and NLR, but not high PLR, was associated with poor response to neoadjuvant anti-HER2 therapy. Furthermore, the combination of high MLR and high NLR enabled better identification of patients with poor responses to anti-HER2 therapy than high MLR or NLR alone.

## Data Availability

The original contributions presented in the study are included in the article/**Supplementary Material**. Further inquiries can be directed to the corresponding authors.
