# Supplementary material for: Association of inflammatory blood markers and pathological complete response in HER2-positive breast cancer: a retrospective single-center cohort study
Source: Front Immunol. 2024 Nov 19;15:1465862. doi: 10.3389/fimmu.2024.1465862 (PMC11611895; doi:10.3389/fimmu.2024.1465862)
Supplement: Supplementary file 1 [file DataSheet1.pdf]

## Supplementary materials

## Supplementary Figure legends

**Figure S1.** Scatter with bar plot of MLR distribution across different populations.

**Figure S2.** Scatter with bar plot of NLR distribution across different populations.

**Figure S3.** Scatter with bar plot of PLR distribution across different populations.

**Figure S1**

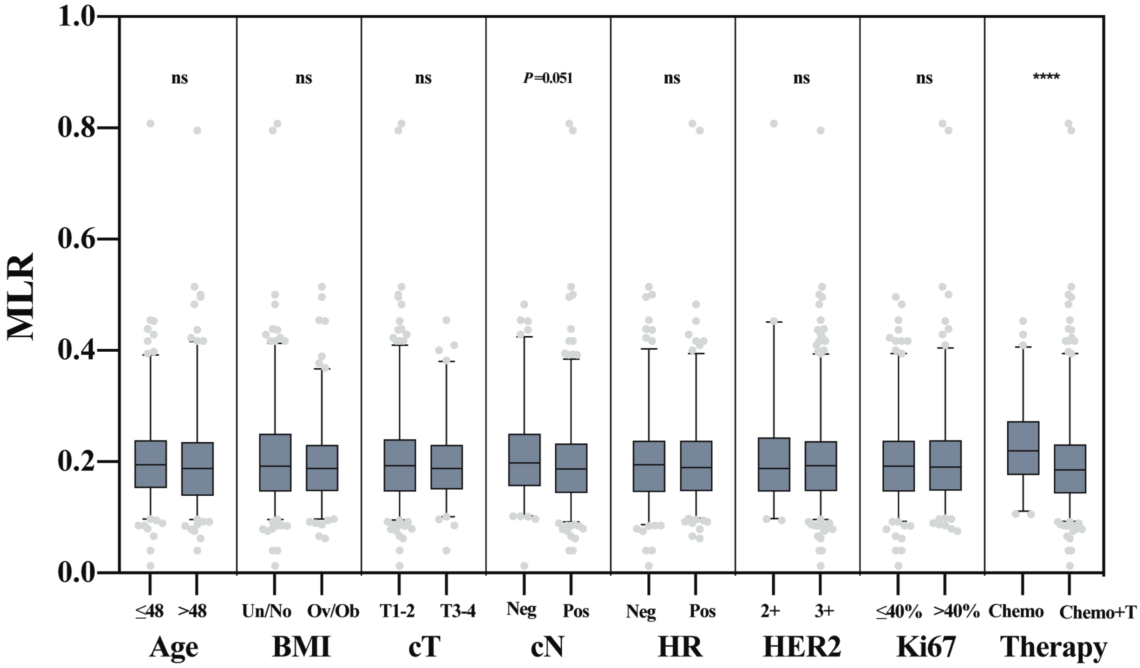

Figure S2

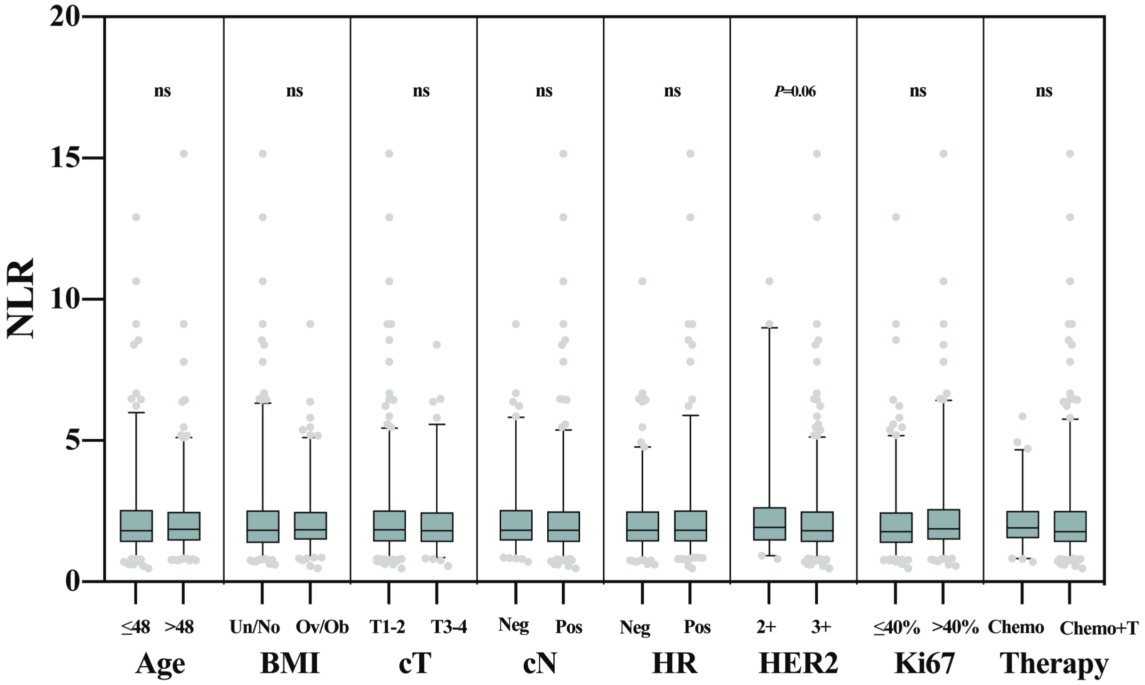


Figure S3

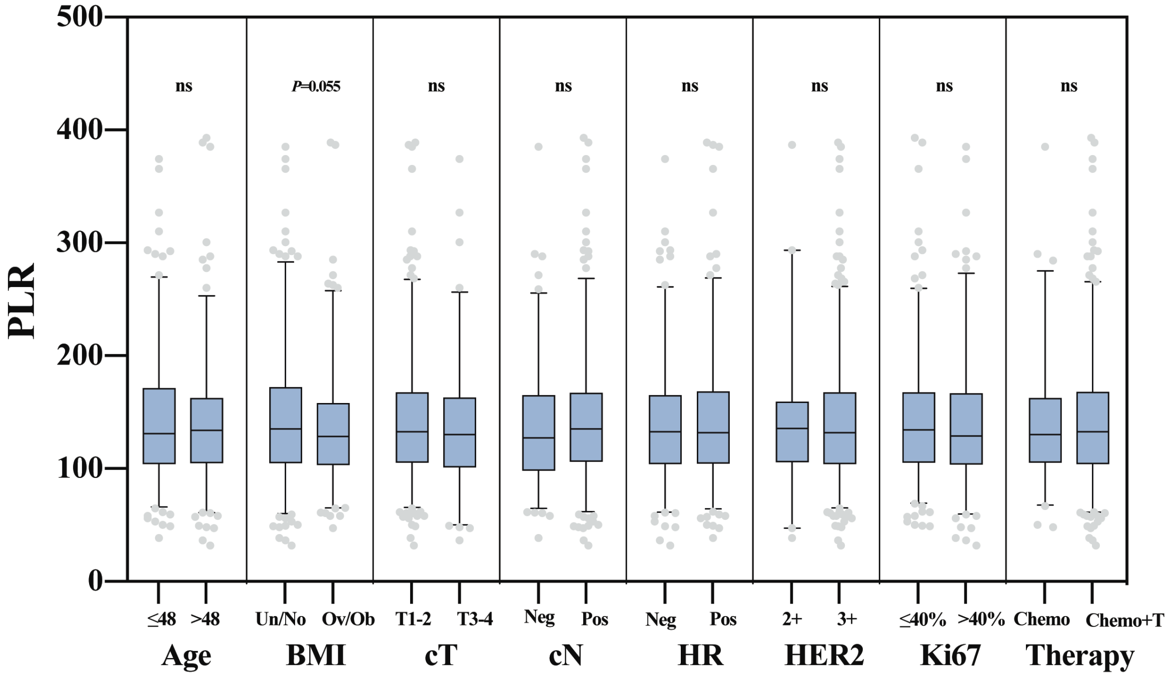

**Supplementary Table 1. Patient characteristics by MLR categories before and after PSM.**

| | Pre-match population | | | After PSM | | |
|---|---|---|---|---|---|---|
| **Characteristics** | **Low (n=372)** | **High (n=372)** | ***P* value** | **Low (n=313)** | **High (n=313)** | **PS adjusted *P*** |
| **Age (years)** | | | 0.379 | | | 0.689 |
| ≤48 | 186 | 198 | | 166 | 171 | |
| >48 | 186 | 174 | | 147 | 142 | |
| **BMI** | | | 0.702 | | | 0.079 |
| Under/Normal weight | 227 | 232 | | 192 | 213 | |
| Overweight/Obesity | 145 | 140 | | 121 | 100 | |
| **Clinical T stage** | | | 0.485 | | | 0.387 |
| T1-2 | 283 | 291 | | 248 | 239 | |
| T3-4 | 89 | 81 | | 65 | 74 | |
| **Nodal status** | | | **0.046** | | | **0.537** |
| Negative | 100 | 125 | | 87 | 94 | |
| Positive | 272 | 247 | | 226 | 219 | |
| **Hormone receptor** | | | 0.34 | | | 0.522 |
| Negative | 168 | 181 | | 156 | 148 | |
| Positive | 204 | 191 | | 157 | 165 | |
| **HER2 staining intensity** | | | 0.289 | | | 0.69 |
| 2+ and FISH + | 45 | 36 | | 33 | 30 | |
| 3+ | 327 | 336 | | 280 | 283 | |
| **Ki67 (%)** | | | 0.883 | | | 0.81 |
| ≤40 | 203 | 205 | | 169 | 172 | |
| >40 | 169 | 167 | | 144 | 141 | |
| **Neoadjuvant therapy** | | | **<0.001** | | | **1** |
| Chemotherapy | 44 | 91 | | 44 | 44 | |
| Chemo+targeted therapy | 328 | 281 | | 269 | 269 | |

**Supplementary Table 2. Patient characteristics by MLR categories before and after IPTW.**

| | Pre-match population | | | | After IPTW | | | |
|---|---|---|---|---|---|---|---|---|
| **Characteristics** | **Low (n=372)** | **High (n=372)** | ***P* value** | **SMD** | **Low (%)** | **High (%)** | **IPTW adjusted *P*** | **SMD** |
| **Age (years)** | | | 0.379 | 0.065 | | | 0.967 | 0.003 |
| ≤48 | 186 | 198 | | | 52 | 51.8 | | |
| >48 | 186 | 174 | | | 48 | 48.2 | | |
| **BMI** | | | 0.702 | 0.028 | | | 0.957 | 0.004 |
| Under/Normal weight | 227 | 232 | | | 62.3 | 62.2 | | |
| Overweight/Obesity | 145 | 140 | | | 37.7 | 37.8 | | |
| **Clinical T stage** | | | 0.485 | 0.051 | | | 0.977 | 0.002 |
| T1-2 | 283 | 291 | | | 77 | 76.9 | | |
| T3-4 | 89 | 81 | | | 23 | 23.1 | | |
| **Nodal status** | | | **0.046** | 0.147 | | | **0.977** | 0.002 |
| Negative | 100 | 125 | | | 30.4 | 30.3 | | |
| Positive | 272 | 247 | | | 69.6 | 69.7 | | |
| **Hormone receptor** | | | 0.34 | 0.07 | | | 0.968 | 0.003 |
| Negative | 168 | 181 | | | 46.8 | 47 | | |
| Positive | 204 | 191 | | | 53.2 | 53 | | |
| **HER2 staining intensity** | | | 0.289 | 0.078 | | | 0.993 | 0.001 |
| 2+ and FISH + | 45 | 36 | | | 11.3 | 11.3 | | |
| 3+ | 327 | 336 | | | 88.7 | 88.7 | | |
| **Ki67 (%)** | | | 0.883 | 0.011 | | | 0.996 | <0.001 |
| ≤40 | 203 | 205 | | | 54.4 | 54.4 | | |
| >40 | 169 | 167 | | | 45.6 | 45.6 | | |
| **Neoadjuvant therapy** | | | **<0.001** | 0.332 | | | **0.985** | 0.01 |
| Chemotherapy | 44 | 91 | | | 18.1 | 18.1 | | |
| Chemo+targeted therapy | 328 | 281 | | | 81.9 | 81.9 | | |

**Supplementary Table 3. Patient characteristics by NLR categories before and after PSM.**

| | Pre-match population | | | After PSM | | |
|---|---|---|---|---|---|---|
| **Characteristics** | **Low (n=372)** | **High (n=372)** | ***P* value** | **Low (n=338)** | **High (n=338)** | **PS adjusted *P*** |
| **Age (years)** | | | 0.463 | | | 0.758 |
| ≤48 | 197 | 187 | | 174 | 170 | |
| >48 | 175 | 185 | | 164 | 168 | |
| **BMI** | | | 0.94 | | | 0.937 |
| Under/Normal weight | 230 | 229 | | 204 | 203 | |
| Overweight/Obesity | 142 | 143 | | 134 | 135 | |
| **Clinical T stage** | | | 0.727 | | | 0.856 |
| T1-2 | 285 | 289 | | 260 | 258 | |
| T3-4 | 87 | 83 | | 78 | 80 | |
| **Nodal status** | | | 0.936 | | | 0.867 |
| Negative | 112 | 113 | | 104 | 102 | |
| Positive | 260 | 259 | | 234 | 236 | |
| **Hormone receptor** | | | 0.826 | | | 0.939 |
| Negative | 173 | 176 | | 160 | 161 | |
| Positive | 199 | 196 | | 178 | 177 | |
| **HER2 staining intensity** | | | 0.126 | | | 0.801 |
| 2+ and FISH + | 34 | 47 | | 34 | 36 | |
| 3+ | 338 | 325 | | 304 | 302 | |
| **Ki67 (%)** | | | 0.185 | | | 0.7 |
| ≤40 | 213 | 195 | | 182 | 177 | |
| >40 | 159 | 177 | | 156 | 161 | |
| **Neoadjuvant therapy** | | | **0.046** | | | **0.601** |
| Chemotherapy | 57 | 78 | | 57 | 52 | |
| Chemo+targeted therapy | 315 | 294 | | 281 | 286 | |

**Supplementary Table 4. Patient characteristics by NLR categories before and after IPTW.**

| | Pre-match population | | | | After IPTW | | | |
|---|---|---|---|---|---|---|---|---|
| **Characteristics** | **Low (n=372)** | **High (n=372)** | ***P* value** | **SMD** | **Low (%)** | **High (%)** | **IPTW adjusted *P*** | **SMD** |
| **Age (years)** | | | 0.463 | 0.054 | | | 0.984 | 0.001 |
| ≤48 | 197 | 187 | | | 51.6 | 51.7 | | |
| >48 | 175 | 185 | | | 48.4 | 48.3 | | |
| **BMI** | | | 0.94 | 0.006 | | | 0.997 | <0.001 |
| Under/Normal weight | 230 | 229 | | | 61.7 | 61.7 | | |
| Overweight/Obesity | 142 | 143 | | | 38.3 | 38.3 | | |
| **Clinical T stage** | | | 0.727 | 0.026 | | | 0.988 | 0.001 |
| T1-2 | 285 | 289 | | | 77.1 | 77.1 | | |
| T3-4 | 87 | 83 | | | 22.9 | 22.9 | | |
| **Nodal status** | | | 0.936 | 0.006 | | | 0.992 | 0.001 |
| Negative | 112 | 113 | | | 30.2 | 30.3 | | |
| Positive | 260 | 259 | | | 69.8 | 69.7 | | |
| **Hormone receptor** | | | 0.826 | 0.016 | | | 0.981 | 0.002 |
| Negative | 173 | 176 | | | 47.3 | 47.2 | | |
| Positive | 199 | 196 | | | 52.7 | 52.8 | | |
| **HER2 staining intensity** | | | 0.126 | 0.112 | | | 0.997 | <0.001 |
| 2+ and FISH + | 34 | 47 | | | 10.9 | 10.9 | | |
| 3+ | 338 | 325 | | | 89.1 | 89.1 | | |
| **Ki67 (%)** | | | 0.185 | 0.097 | | | 0.995 | <0.001 |
| ≤40 | 213 | 195 | | | 54.9 | 54.8 | | |
| >40 | 159 | 177 | | | 45.1 | 45.2 | | |
| **Neoadjuvant therapy** | | | **0.046** | 0.147 | | | **0.991** | 0.001 |
| Chemotherapy | 57 | 78 | | | 18.1 | 18.1 | | |
| Chemo+targeted therapy | 315 | 294 | | | 81.9 | 81.9 | | |

**Supplementary Table 5. Patient characteristics by PLR categories before and after PSM.**

| | Pre-match population | | | After PSM | | |
|---|---|---|---|---|---|---|
| **Characteristics** | **Low (n=372)** | **High (n=372)** | ***P* value** | **Low (n=344)** | **High (n=344)** | **PS adjusted *P*** |
| **Age (years)** | | | 0.66 | | | 1 |
| ≤48 | 195 | 189 | | 180 | 180 | |
| >48 | 177 | 183 | | 164 | 164 | |
| **BMI** | | | 0.258 | | | 0.876 |
| Under/Normal weight | 222 | 237 | | 209 | 211 | |
| Overweight/Obesity | 150 | 135 | | 135 | 133 | |
| **Clinical T stage** | | | 0.861 | | | 0.527 |
| T1-2 | 286 | 288 | | 261 | 268 | |
| T3-4 | 86 | 84 | | 83 | 76 | |
| **Nodal status** | | | **0.031** | | | **0.32** |
| Negative | 126 | 99 | | 111 | 99 | |
| Positive | 246 | 273 | | 233 | 245 | |
| **Hormone receptor** | | | 0.826 | | | 0.593 |
| Negative | 173 | 176 | | 159 | 166 | |
| Positive | 199 | 196 | | 185 | 178 | |
| **HER2 staining intensity** | | | 0.556 | | | 0.71 |
| 2+ and FISH + | 38 | 43 | | 38 | 35 | |
| 3+ | 334 | 329 | | 306 | 309 | |
| **Ki67 (%)** | | | 0.302 | | | 0.819 |
| ≤40 | 197 | 211 | | 186 | 183 | |
| >40 | 175 | 161 | | 158 | 161 | |
| **Neoadjuvant therapy** | | | 0.634 | | | 0.846 |
| Chemotherapy | 70 | 65 | | 66 | 64 | |
| Chemo+targeted therapy | 302 | 307 | | 278 | 280 | |

**Supplementary Table 6. Patient characteristics by PLR categories before and after IPTW.**

| | Pre-match population | | | | After IPTW | | | |
|---|---|---|---|---|---|---|---|---|
| **Characteristics** | **Low (n=372)** | **High (n=372)** | ***P* value** | **SMD** | **Low (%)** | **High (%)** | **IPTW adjusted *P*** | **SMD** |
| **Age (years)** | | | 0.66 | 0.032 | | | 0.994 | 0.001 |
| ≤48 | 195 | 189 | | | 51.5 | 51.5 | | |
| >48 | 177 | 183 | | | 48.5 | 48.5 | | |
| **BMI** | | | 0.258 | 0.083 | | | 0.995 | 0.001 |
| Under/Normal weight | 222 | 237 | | | 61.5 | 61.5 | | |
| Overweight/Obesity | 150 | 135 | | | 38.5 | 38.5 | | |
| **Clinical T stage** | | | 0.861 | 0.013 | | | 0.991 | 0.001 |
| T1-2 | 286 | 288 | | | 77.1 | 77.1 | | |
| T3-4 | 86 | 84 | | | 22.9 | 22.9 | | |
| **Nodal status** | | | **0.031** | 0.159 | | | **0.99** | 0.001 |
| Negative | 126 | 99 | | | 30.4 | 30.5 | | |
| Positive | 246 | 273 | | | 69.6 | 69.5 | | |
| **Hormone receptor** | | | 0.826 | 0.016 | | | 0.989 | 0.001 |
| Negative | 173 | 176 | | | 47.1 | 47 | | |
| Positive | 199 | 196 | | | 52.9 | 53 | | |
| **HER2 staining intensity** | | | 0.556 | 0.043 | | | 0.979 | 0.002 |
| 2+ and FISH + | 38 | 43 | | | 11 | 11 | | |
| 3+ | 334 | 329 | | | 89 | 89 | | |
| **Ki67 (%)** | | | 0.302 | 0.076 | | | 0.999 | <0.001 |
| ≤40 | 197 | 211 | | | 54.5 | 54.5 | | |
| >40 | 175 | 161 | | | 45.5 | 45.5 | | |
| **Neoadjuvant therapy** | | | 0.634 | 0.035 | | | 0.998 | <0.001 |
| Chemotherapy | 70 | 65 | | | 18.2 | 18.2 | | |
| Chemo+targeted therapy | 302 | 307 | | | 81.8 | 81.8 | | |

**Supplementary Table 7. Multivariable analysis of the relationship between MLR and pCR before and after matching.**

| | Pre-match population | | | After PSM | | | After IPTW | | |
|---|---|---|---|---|---|---|---|---|---|
| **Characteristics** | **OR** | **95%CI** | ***P* value** | **OR** | **95%CI** | ***P* value** | **OR** | **95%CI** | ***P* value** |
| **MLR** | | | | | | | | | |
| Low | 1 | | | 1 | | | 1 | | |
| High | 0.802 | 0.580-1.108 | 0.18 | 0.811 | 0.573-1.149 | 0.239 | 0.813 | 0.589-1.124 | 0.211 |
| **Age (years)** | | | | | | | | | |
| ≤48 | 1 | | | 1 | | | 1 | | |
| >48 | 1.285 | 0.928-1.780 | 0.132 | 1.193 | 0.839-1.696 | 0.325 | 1.285 | 0.925-1.787 | 0.135 |
| **BMI** | | | | | | | | | |
| Under/Normal weight | 1 | | | 1 | | | 1 | | |
| Overweight/Obesity | 0.587 | 0.417-0.825 | **0.002** | 0.513 | 0.352-0.746 | **<0.001** | 0.585 | 0.414-0.828 | **0.002** |
| **Clinical T stage** | | | | | | | | | |
| T1-2 | 1 | | | 1 | | | 1 | | |
| T3-4 | 0.832 | 0.566-1.221 | 0.347 | 0.872 | 0.569-1.334 | 0.527 | 0.846 | 0.574-1.247 | 0.399 |
| **Nodal status** | | | | | | | | | |
| Negative | 1 | | | 1 | | | 1 | | |
| Positive | 1.028 | 0.708-1.493 | 0.884 | 1.014 | 0.679-1.515 | 0.945 | 1.005 | 0.689-1.467 | 0.979 |
| **Hormone receptor** | | | | | | | | | |
| Negative | 1 | | | 1 | | | 1 | | |
| Positive | 0.473 | 0.341-0.655 | **<0.001** | 0.453 | 0.318-0.646 | **<0.001** | 0.444 | 0.319-0.617 | **<0.001** |
| **HER2 staining intensity** | | | | | | | | | |
| 2+ and FISH + | 1 | | | 1 | | | 1 | | |
| 3+ | 3.172 | 1.599-6.291 | **0.001** | 2.422 | 1.158-5.066 | **0.019** | 2.954 | 1.502-5.808 | **0.002** |
| **Ki67 (%)** | | | | | | | | | |
| ≤40 | 1 | | | 1 | | | 1 | | |
| >40 | 1.917 | 1.386-2.653 | **<0.001** | 2.117 | 1.486-3.014 | **<0.001** | 1.938 | 1.395-2.693 | **<0.001** |
| **Neoadjuvant therapy** | | | | | | | | | |
| Chemotherapy | 1 | | | 1 | | | 1 | | |
| Chemo+targeted therapy | 4.406 | 2.549-7.617 | **<0.001** | 5.33 | 2.667-10.651 | **<0.001** | 6.097 | 3.367-11.043 | **<0.001** |

**Supplementary Table 8. Multivariable analysis of the relationship between NLR and pCR before and after matching.**

| Characteristics | Pre-match population | | | After PSM | | | After IPTW | | |
|---|---|---|---|---|---|---|---|---|---|
| | OR | 95%CI | *P* value | OR | 95%CI | *P* value | OR | 95%CI | *P* value |
| **NLR** | | | | | | | | | |
| Low | 1 | | | 1 | | | 1 | | |
| High | 0.787 | 0.570-1.085 | 0.144 | 0.789 | 0.565-1.101 | 0.163 | 0.789 | 0.573-1.088 | 0.148 |
| **Age (years)** | | | | | | | | | |
| ≤48 | 1 | | | 1 | | | 1 | | |
| >48 | 1.303 | 0.941-1.805 | 0.111 | 1.302 | 0.927-1.829 | 0.128 | 1.291 | 0.931-1.788 | 0.125 |
| **BMI** | | | | | | | | | |
| Under/Normal weight | 1 | | | 1 | | | 1 | | |
| Overweight/Obesity | 0.585 | 0.416-0.822 | **0.002** | 0.627 | 0.441-0.892 | **0.009** | 0.597 | 0.424-0.840 | **0.003** |
| **Clinical T stage** | | | | | | | | | |
| T1-2 | 1 | | | 1 | | | 1 | | |
| T3-4 | 0.836 | 0.569-1.227 | 0.36 | 0.844 | 0.567-1.257 | 0.403 | 0.85 | 0.579-1.249 | 0.409 |
| **Nodal status** | | | | | | | | | |
| Negative | 1 | | | 1 | | | 1 | | |
| Positive | 1.036 | 0.714-1.505 | 0.852 | 1.054 | 0.714-1.555 | 0.793 | 1.036 | 0.712-1.506 | 0.855 |
| **Hormone receptor** | | | | | | | | | |
| Negative | 1 | | | 1 | | | 1 | | |
| Positive | 0.474 | 0.342-0.657 | **<0.001** | 0.461 | 0.329-0.647 | **<0.001** | 0.479 | 0.345-0.664 | **<0.001** |
| **HER2 staining intensity** | | | | | | | | | |
| 2+ and FISH + | 1 | | | 1 | | | 1 | | |
| 3+ | 3.09 | 1.555-6.140 | **0.001** | 3.184 | 1.550-6.539 | **0.002** | 3.259 | 1.629-6.519 | **0.001** |
| **Ki67 (%)** | | | | | | | | | |
| ≤40 | 1 | | | 1 | | | 1 | | |
| >40 | 1.941 | 1.402-2.687 | **<0.001** | 1.919 | 1.366-2.694 | **<0.001** | 1.939 | 1.4-2.685 | **<0.001** |
| **Neoadjuvant therapy** | | | | | | | | | |
| Chemotherapy | 1 | | | 1 | | | 1 | | |
| Chemo+targeted therapy | 4.528 | 2.625-7.811 | **<0.001** | 4.41 | 2.438-7.978 | **<0.001** | 4.929 | 2.828-8.592 | **<0.001** |

**Supplementary Table 9. Multivariable analysis of the relationship between PLR and pCR before and after matching.**

| | Pre-match population | | | After PSM | | | After IPTW | | |
|---|---|---|---|---|---|---|---|---|---|
| **Characteristics** | **OR** | **95%CI** | ***P* value** | **OR** | **95%CI** | ***P* value** | **OR** | **95%CI** | ***P* value** |
| **PLR** | | | | | | | | | |
| Low | 1 | | | 1 | | | 1 | | |
| High | 0.939 | 0.681-1.294 | 0.699 | 0.981 | 0.704-1.368 | 0.909 | 0.938 | 0.681-1.291 | 0.694 |
| **Age (years)** | | | | | | | | | |
| ≤48 | 1 | | | 1 | | | 1 | | |
| >48 | 1.295 | 0.935-1.792 | 0.119 | 1.354 | 0.965-1.901 | 0.079 | 1.296 | 0.936-1.794 | 0.118 |
| **BMI** | | | | | | | | | |
| Under/Normal weight | 1 | | | 1 | | | 1 | | |
| Overweight/Obesity | 0.587 | 0.418-0.826 | **0.002** | 0.551 | 0.388-0.784 | **0.001** | 0.574 | 0.409-0.807 | **0.001** |
| **Clinical T stage** | | | | | | | | | |
| T1-2 | 1 | | | 1 | | | 1 | | |
| T3-4 | 0.839 | 0.572-1.231 | 0.37 | 0.81 | 0.545-1.206 | 0.3 | 0.855 | 0.583-1.253 | 0.421 |
| **Nodal status** | | | | | | | | | |
| Negative | 1 | | | 1 | | | 1 | | |
| Positive | 1.042 | 0.717-1.514 | 0.83 | 1.087 | 0.739-1.600 | 0.671 | 1.024 | 0.706-1.486 | 0.899 |
| **Hormone receptor** | | | | | | | | | |
| Negative | 1 | | | 1 | | | 1 | | |
| Positive | 0.478 | 0.345-0.661 | **<0.001** | 0.461 | 0.329-0.647 | **<0.001** | 0.465 | 0.336-0.644 | **<0.001** |
| **HER2 staining intensity** | | | | | | | | | |
| 2+ and FISH + | 1 | | | 1 | | | 1 | | |
| 3+ | 3.147 | 1.586-6.245 | **0.001** | 2.675 | 1.329-5.384 | **0.006** | 2.815 | 1.448-5.471 | **0.002** |
| **Ki67 (%)** | | | | | | | | | |
| ≤40 | 1 | | | 1 | | | 1 | | |
| >40 | 1.907 | 1.378-2.638 | **<0.001** | 1.787 | 1.279-2.497 | **0.001** | 1.902 | 1.375-2.629 | **<0.001** |
| **Neoadjuvant therapy** | | | | | | | | | |
| Chemotherapy | 1 | | | 1 | | | 1 | | |
| Chemo+targeted therapy | 4.601 | 2.671-7.925 | **<0.001** | 4.391 | 2.542-7.588 | **<0.001** | 4.711 | 2.731-8.126 | **<0.001** |

**Supplementary Table 10. Multivariable analysis of the relationship between MLR/NLR combination and pCR in overall population.**

| | Multivariable analysis | | |
|---|---|---|---|
| **Characteristics** | **OR** | **95%CI** | ***P* value** |
| **MLR/NLR** | | | |
| MLR/NLR (Low/Low) | 1 | | |
| MLR/NLR (High/Low or Low/High) | 1.085 | 0.730-1.612 | 0.686 |
| MLR/NLR (High/High) | 0.714 | 0.484-1.052 | 0.089 |
| **Age (years)** | | | |
| ≤48 | 1 | | |
| >48 | 1.303 | 0.940-1.807 | 0.112 |
| **BMI** | | | |
| Under/Normal weight | 1 | | |
| Overweight/Obesity | 0.577 | 0.410-0.813 | **0.002** |
| **Clinical T stage** | | | |
| T1-2 | 1 | | |
| T3-4 | 0.821 | 0.558-1.206 | 0.315 |
| **Nodal status** | | | |
| Negative | 1 | | |
| Positive | 1.049 | 0.721-1.525 | 0.803 |
| **Hormone receptor** | | | |
| Negative | 1 | | |
| Positive | 0.474 | 0.342-0.658 | **<0.001** |
| **HER2 staining intensity** | | | |
| 2+ and FISH + | 1 | | |
| 3+ | 3.248 | 1.632-6.467 | **0.001** |
| **Ki67 (%)** | | | |
| ≤40 | 1 | | |
| >40 | 1.935 | 1.397-2.681 | **<0.001** |
| **Neoadjuvant therapy** | | | |
| Chemotherapy | 1 | | |
| Chemo+targeted therapy | 4.382 | 2.536-7.572 | **<0.001** |

**Supplementary Table 11. Multivariable analysis of the relationship between MLR/NLR combination and pCR in patients treated with chemotherapy alone.**

| | Multivariable analysis | | |
|---|---|---|---|
| **Characteristics** | **OR** | **95%CI** | ***P* value** |
| **MLR/NLR** | | | |
| MLR/NLR (Low/Low) | 1 | | |
| MLR/NLR (High/Low or Low/High) | 2.554 | 0.242-26.992 | 0.436 |
| MLR/NLR (High/High) | 9.528 | 1.154-78.638 | **0.036** |
| **Age (years)** | | | |
| ≤48 | 1 | | |
| >48 | 1.057 | 0.345-3.241 | 0.923 |
| **BMI** | | | |
| Under/Normal weight | 1 | | |
| Overweight/Obesity | 0.493 | 0.154-1.580 | 0.234 |
| **Clinical T stage** | | | |
| T1-2 | 1 | | |
| T3-4 | 0.818 | 0.212-3.162 | 0.771 |
| **Nodal status** | | | |
| Negative | 1 | | |
| Positive | 0.908 | 0.291-2.832 | 0.868 |
| **Hormone receptor** | | | |
| Negative | 1 | | |
| Positive | 1.512 | 0.492-4.645 | 0.47 |
| **HER2 staining intensity** | | | |
| 2+ and FISH + | 1 | | |
| 3+ | 4.753 | 0.533-42.423 | 0.163 |
| **Ki67 (%)** | | | |
| ≤40 | 1 | | |
| >40 | 1.448 | 0.497-4.218 | 0.497 |

**Supplementary Table 12. Multivariable analysis of the relationship between MLR/NLR combination and pCR in patients treated with chemotherapy plus anti-HER2 therapy.**

| | Multivariable analysis | | |
|---|---|---|---|
| **Characteristics** | **OR** | **95%CI** | ***P* value** |
| **MLR/NLR** | | | |
| MLR/NLR (Low/Low) | 1 | | |
| MLR/NLR (High/Low or Low/High) | 1.077 | 0.708-1.638 | 0.728 |
| MLR/NLR (High/High) | 0.548 | 0.361-0.832 | **0.005** |
| **Age (years)** | | | |
| ≤48 | 1 | | |
| >48 | 1.343 | 0.946-1.907 | 0.099 |
| **BMI** | | | |
| Under/Normal weight | 1 | | |
| Overweight/Obesity | 0.575 | 0.398-0.829 | **0.003** |
| **Clinical T stage** | | | |
| T1-2 | 1 | | |
| T3-4 | 0.835 | 0.551-1.266 | 0.396 |
| **Nodal status** | | | |
| Negative | 1 | | |
| Positive | 1.054 | 0.700-1.586 | 0.801 |
| **Hormone receptor** | | | |
| Negative | 1 | | |
| Positive | 0.405 | 0.286-0.575 | **<0.001** |
| **HER2 staining intensity** | | | |
| 2+ and FISH + | 1 | | |
| 3+ | 3.24 | 1.559-6.734 | **0.002** |
| **Ki67 (%)** | | | |
| ≤40 | 1 | | |
| >40 | 2.043 | 1.437-2.903 | **<0.001** |
